# The Use of Mixed Methods Research Methods (MMR) in Cognitive Impairment and Dementia: A Review

**DOI:** 10.1192/j.eurpsy.2026.11562

**Published:** 2026-07-31

**Authors:** S. Yıldırım

**Affiliations:** 1 Centre for Mind/Brain Sciences, University of Trento, Trento, Italy; 2 Institute of Social Sciences, Ankara Yıldırım Beyazıt University, Ankara, Türkiye

## Abstract

**Introduction:**

Understanding cognitive impairment and dementia requires both quantitative precision and qualitative depth. Mixed Methods Research (MMR) offers a framework that combines these approaches to capture clinical outcomes alongside lived experiences. Despite its increasing use in health sciences, the specific application and methodological quality of MMR in dementia-related studies remain underexplored.

**Objectives:**

This review aimed to systematically evaluate MMR studies on cognitive impairment and dementia, focusing on their design typologies, data integration strategies, methodological rigor, and contribution to the field.

**Methods:**

Following the PRISMA 2020 framework, a systematic search was conducted across PubMed and ProQuest databases for studies published in 2025. Inclusion criteria were peer-reviewed articles explicitly using MMR and focusing on dementia or cognitive impairment. Ten eligible studies were evaluated in terms of design (sequential, convergent, embedded, or hybrid), rationale, data collection, and integration levels.

**Results:**

The majority of studies were intervention-based, aiming to improve cognitive rehabilitation, care models, or digital support systems. Quantitative components often dominated the analysis, while qualitative findings enriched contextual understanding. However, integration between data types was generally weak, often confined to the discussion stage. Most studies were multi-authored, publicly funded, and involved diverse participant groups including patients, caregivers, and healthcare professionals.

**Conclusions:**

MMR provides a powerful framework for cognitive impairment and dementia research by enabling multidimensional insights into clinical and psychosocial aspects. Yet, limited integration between qualitative and quantitative data reduces its full potential. Strengthening methodological transparency and interdisciplinary collaboration will enhance the quality and applicability of future MMR studies in cognitive impairment and dementia.

**Keywords:**

Cognitive impairment, dementia, mixed methods research

**Disclosure of Interest:**

None Declared

